# Logarithmic coding leads to adaptive stabilization in the presence of sensorimotor delays

**DOI:** 10.1073/pnas.2510385122

**Published:** 2025-10-23

**Authors:** Leonardo Demarchi, Monica Coraggioso, Antoine Hubert, Thomas Panier, Ghislaine Morvan-Dubois, Volker Bormuth, Georges Debrégeas

**Affiliations:** ^a^Sorbonne Université, CNRS, Laboratoire Jean Perrin, Paris F75005, France; ^b^Sorbonne Université, CNRS, INSERM, Institut de Biologie Paris-Seine, Paris F75005, France

**Keywords:** sensorimotor control, *Danionella cerebrum*, optical flow stabilization, whole-brain imaging, delay-induced oscillations

## Abstract

Animals respond to sensory stimuli with motor actions, which in turn generate new sensory inputs. This sensorimotor loop is constrained by time delays that impose a trade-off between responsiveness and stability. Additionally, as the relationship between a motor command and the corresponding sensory feedback is context-dependent, the response must be adapted in real time. It is generally believed that this adaptation process relies on an internal model that is continuously updated through prediction error minimization. Here, we experimentally reveal an alternative strategy based on a simpler feedback mechanism that does not require any internal model. We developed a virtual reality system for the miniature transparent fish *Danionella cerebrum* that enables in vivo brain-wide imaging during fictive navigation. By systematically manipulating the feedback parameters, we dissected the motor control process that allows the animal to stabilize its position using optic flow. The sensorimotor loop can be fully described by a single delay differential equation, whose solutions quantitatively capture the observed behavior across all experimental conditions. Both behavioral and neural data indicate that the observed adaptive response arises from logarithmic nonlinearities at the sensory (Weber–Fechner law) and motor (Henneman’s size principle) ends. These fundamental properties of the nervous system, conserved across species and sensory modalities, have traditionally been interpreted in terms of efficient coding. Our findings unveil a distinct functional role for such nonlinear transformations: ensuring stability in sensorimotor control despite inherent delays and sensory uncertainty.

## Significance

Animal movement is shaped by the continuous interaction between motor actions and sensory feedback. This process is challenging due to time delays, which can have destabilizing effects, and the constantly varying relationship between motor commands and sensory feedback. Identifying adaptive strategies that ensure both stability and performance, and their implementation in the brain, remains a central challenge in neuroscience. We address this by simultaneously monitoring behavior and brain activity in the miniature transparent fish *Danionella cerebrum* during visually guided navigation in a virtual reality system. Using a dynamical systems approach, we find that their adaptive response arises from nonlinear encoding of both sensory and motor information, revealing a distinct functional role for these fundamental properties of the nervous system.

Animals continuously interact with their environment through sensorimotor loops. These dynamic processes are inherently constrained by finite time delays and noise in the sensory and motor systems which can lead to instability (1, 2). Moreover, the relationship between motor commands and the resulting sensory feedback is not fixed but rather depends on the state of both the body and the external world. For a given context, there exists an optimal response function that maximizes behavioral performance (3). As the context varies, animals need to adapt their response in a flexible manner (4).

A key example is optic flow navigation, where animals use visual cues to estimate their motion in order to maintain an intended trajectory (5, 6). This behavior is ubiquitous among motile species, but it is particularly important for flying and swimming animals, which can be easily thrown off course by external currents. For translational motion, the relationship between the animal speed and the retinal optic flow is ambiguous, as it depends on the distance of the animal from the surrounding objects, a parameter that varies in time and cannot be directly sensed by the animal (7).

To cope with such challenges, some animals use forward models to internally predict the sensory consequences of their motor commands, thereby distinguishing them from external perturbations (4, 8, 9). These internal models may be either hardwired or constantly updated by minimizing prediction errors, enabling the system to adapt to a changing context through motor learning. Moreover, predictive processing can help mitigate the destabilizing effects of feedback delays. Although internal models provide a powerful framework for efficient motor control (10–12), it remains unclear whether simpler mechanisms are employed by animals to address the same challenges.

We address this question using the miniature freshwater fish *Danionella cerebrum*, a recently introduced model vertebrate whose size and transparency enables in vivo whole-brain imaging across all developmental stages (13, 14). We first use a virtual reality system to dissect the sensorimotor computation at play during optic flow navigation. We then use whole-brain imaging to probe the dynamics of the neuronal population engaged in this process.

## Results

### Stabilization of Visual Flows in a Virtual Reality System.

**Fig. 1.**
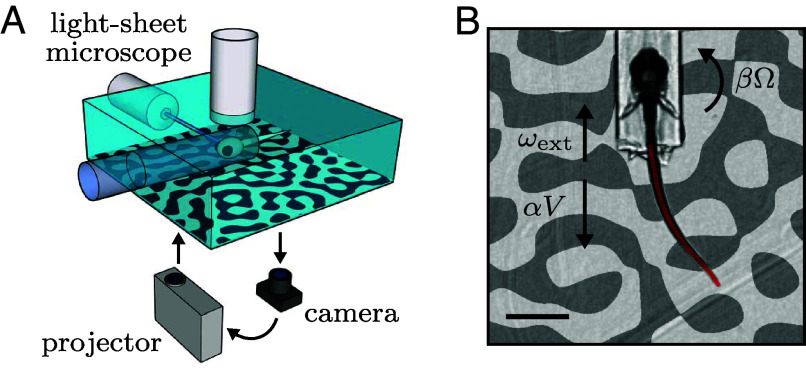
Virtual reality system. (*A*) Schematic of the experimental setup. Fish are head-tethered in agarose. Their tail movements are video-monitored and a projector displays a visual pattern on the opaque bottom of the tank. Brain-imaging can be simultaneously performed using a light-sheet microscope. (*B*) Snapshot of a fictively swimming fish. From successive tail profiles (red line), we extract the fictive speed V, and angular velocity Ω which are used to compute the translational and rotational feedback flow rates αV and βΩ, respectively. We additionally simulate an external current with flow rate $\omega_{\text{ext}}$. (Scale bar, 1 mm.).

The fish were head-tethered using agarose, then placed in a virtual-reality setup that comprised a camera to monitor tail movement, a projector to display visual patterns beneath the animal, and a light-sheet microscope for brain imaging (Fig. 1*A*, *Materials and Methods*). Experiments were performed on two-week-old danionella, which, unlike zebrafish larvae, exhibit continuous swimming in both freely moving and head-tethered configurations, with a quasi-constant tail-beat frequency of ≈16 Hz (15). Using a fluid dynamics model calibrated against freely swimming recordings, we inferred the fictive forward speed V and angular velocity of the fish in real-time from the video-monitored tail movement (16, 17) (Fig. 1*B*, *Materials and Methods*). These values were used to compute the visual feedback, i.e., the translational and rotational velocity of the projected pattern to induce the illusion of self-motion in the fish.

**Fig. 2.**
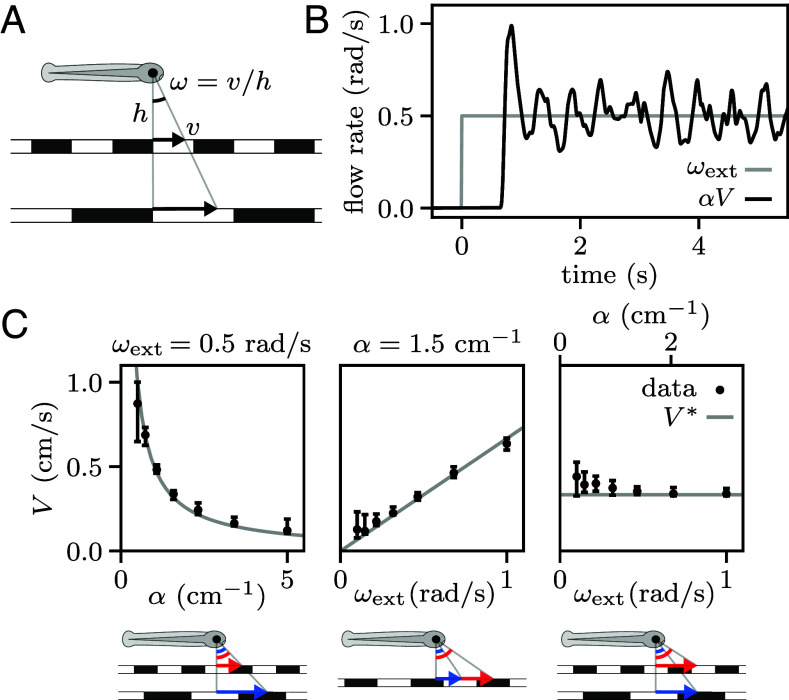
Fish adapt their swimming speed to stabilize external visual flows. (*A*) Definition of the optic flow rate ω. Rescaling the pattern size, distance h, and speed v by the same factor does not affect the perceived optic flow. (*B*) Example trace showing the fish response to forward pattern motion. After a short latency, the fish adjusts its swimming speed to match external and feedback flow rates. (*C*) Median swimming speed (black error bars indicate quartiles of the distribution across N=(33,31,28) fish, from left to right) for different values of the external flow rate $\omega_{\text{ext}}$ and feedback gain α. Fish can adapt their swimming speed to the target value (gray lines) to stabilize themselves against currents. The schematics below the graphs illustrate the equivalence of the parameter changes in the real world, with the arrows denoting the speed of the external current for two different parameter values (larger in red).

In the real world, a movement of the fish at a certain speed corresponds, from the animal’s egocentric perspective, to an opposite movement of the environment with the same speed. However, what the animal actually perceives is the resulting optic flow on its retina, which only depends on the ratio between the speed and the distance of the surrounding objects. For a pattern moving at speed v, the optic flow is characterized by the rate ω=v/h, where h is the pattern distance, which in our case is 5mm (Fig. 2*A*). We implemented the feedback by translating the pattern backward, resulting in a flow rate αV, where the feedback gain α can be interpreted as the inverse of the pattern distance in the virtual environment. Note that the ambiguity in relating optic flow and self-motion is specific to translational movement: the angular velocity can be directly inferred from the rotational optic flow. We implemented rotational feedback by rotating the pattern with angular velocity βΩ, where a rotational feedback gain β=1 corresponds to freely swimming conditions.

We elicited an optomotor response by translating the pattern in a certain direction with an imposed rate $\omega_{\text{ext}}$, simulating an external current dragging the fish in the opposite direction (18). The fish responded to such a stimulus by actively turning to align with the fictive current (Movie S1). Here we focus on the case where the external current starts off directed along the fish heading direction, corresponding to a fictive backward movement. If the fish remains approximately aligned with the current then the optic flow is directed along the fish heading with net rate $\omega =\omega_{\text{ext}}-\alpha V$.

The absence of visual feedback (α=0, open-loop) rapidly induces the fish to give up and stop swimming. Otherwise (α>0, closed-loop), they can keep swimming for sustained periods of time and adapt their swimming speed to match external and feedback flow rates such that $V≃V^{*}=\omega_{\text{ext}}/\alpha$ (Fig. 2*B* and Movies S2 and S3). Equivalently, this means that their position in the virtual space remains approximately constant.

We assessed the robustness of this behavior by presenting fish with external currents of varying optic flow rate $\omega_{\text{ext}}$ and for different values of the feedback gain α, in randomized trials lasting 30 s. We considered three experimental paradigms (Fig. 2*C*): fixed external flow rate (varying α with fixed $\omega_{\text{ext}}$), fixed pattern distance (varying $\omega_{\text{ext}}$ with fixed α), fixed current speed (varying $\omega_{\text{ext}}$ and α with a fixed target speed $V^{*}$). We found that most fish adapt their speed to compensate for the external current, provided that the target speed $V^{*}$ falls within a physiologically accessible range (*SI Appendix*, section 1G).

### Sensorimotor Delays Induce Sustained Speed Oscillations.

We noticed that the fictive speed signals displayed quasi-periodic oscillations around the target value $V^{*}$, as illustrated in Fig. 2*B*. These speed modulations have a characteristic frequency of ≈1.5 Hz and an amplitude proportional to the target speed (*SI Appendix*, sections 1H–I). We hypothesized that they could be a signature of delays in the sensorimotor loop. Intuitively, if the correcting action lags the driving signal by a finite delay τ, the fish may systematically overcompensate for the same duration after crossing the target speed, leading to oscillations of period ∼4τ (19).

**Fig. 3.**
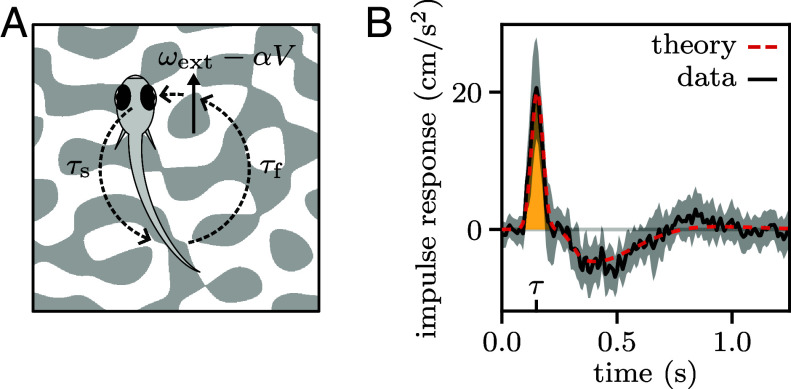
Time delays in the sensorimotor loop. (*A*) Schematic showing the delays in the sensorimotor loop. The fish perceives a certain optic flow and updates its swimming speed after a sensorimotor delay $\tau_{\text{s}}$. A given tail movement induces a change in the optic flow after a feedback delay $\tau_{\text{f}}$. (*B*) Reverse correlation gives an estimate of the impulse response function (black line and gray area, mean and SD over N=11 fish), i.e., the evolution of $\dot{V}$ following an impulse perturbation of $\omega_{\text{ext}}$. The initial peak represents the response kernel (yellow area), whose time gives an estimate of the total delay $\tau =\tau_{\text{s}}+\tau_{\text{f}}$. Integrating Eq. **1** with this response kernel, we obtained the theoretical impulse response shown in red.

We measured the sensorimotor delay by modulating the imposed optic flow with a white-noise signal, while monitoring the fish velocity. The impulse response function, defined as the acceleration signal evoked by an impulse of optic flow rate, was then estimated by reverse correlation (20). This response function exhibits a sharp delayed positive peak, corresponding to the response kernel, followed by a negative modulation reflecting the feedback mechanism (Fig. 3*B*).

The time of the peak provides an estimate of the delay τ=150±20 ms. This value is close to one-fourth of the oscillatory period observed in fish swimming under constant current flow (Fig. 2*B*), supporting our initial hypothesis. This sensorimotor delay can be decomposed into two components, $\tau =\tau_{\text{s}}+\tau_{\text{f}}$, where $\tau_{\text{s}}$ corresponds to the internal delay between the sensory perception and the execution of the correcting motor action, while $\tau_{\text{f}}$ accounts for the lag between the tail motion and the resulting change in optic flow (Fig. 3*A*). While in freely swimming conditions $\tau_{\text{f}}$ is due to inertia, here it corresponds to the latency of the virtual reality, which we measured to be $\tau_{\text{f}}=60\;\pm \;4$ ms (*SI Appendix*, section 1E). Therefore, the sensory-to-motor internal delay is $\tau_{\text{s}}=90\pm 20$ ms, of the order of a tail-beat period. This value was found to be independent of both $\omega_{\text{ext}}$ and α (*SI Appendix*, section 2B).

**Fig. 4.**
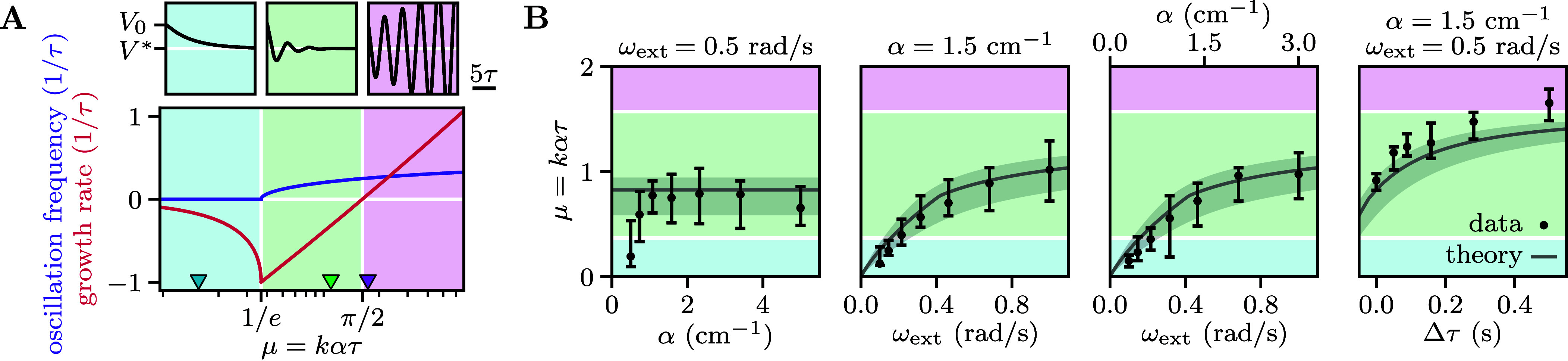
Fish adapt their responsiveness to prevent delay-induced instability. (*A*) Growth rate and oscillation frequency of the solutions to Eq. **1** as a function of the dimensionless gain μ. The three dynamical regimes are illustrated by example trajectories (top plots) for three values of μ (colored triangles). (*B*) Estimated values of the dimensionless gain μ through linear regression [black error bars, quartiles of the distributions over N=(33,31,28,14) fish, from left to right]. The predictions of the effective linear theory of Eq. **2** are shown in gray.

To model the sensorimotor loop, we first assumed that the fish acceleration at time t is proportional to the net flow rate at time t−τ:

[1] $$\begin{array}{c} \dot{V}=k\omega {{|}_{t-\tau }=k\left(\omega_{\text{ext}}-\alpha V\right)|}_{t-\tau } \end{array}$$

where the parameter k denotes the responsiveness of the fish to optic flow. Rewriting Eq. **1** in dimensionless form, we found that the system dynamics are governed by a single dimensionless gain, μ=kατ, whose value defines the stability regime (Fig. 4*A*, *Materials and Methods*). For μ<1/e, V(t) decays exponentially to the target value $V^{*}$. For 1/e<μ<π/2, it converges to $V^{*}$ through damped oscillations. At μ=π/2, one observes a Hopf-like bifurcation, followed by an unstable regime with diverging oscillations. This equation could thus lead to the sustained oscillations observed in our data through two distinct mechanisms: in the intermediate regime, sensorimotor noise could drive the system away from the target speed eliciting noise-induced oscillations; in the unstable regime, nonlinearities could prevent the system from diverging leading to limit cycle oscillations.

This simple linear model accounts for nontrivial aspects of the system dynamics. We forced speed oscillations with a sinusoidal modulation of the external flow. Using the model, we could quantitatively reproduce the observed phase lag between the fish speed oscillations and the driving signal over a wide range of driving frequencies (*SI Appendix*, section 2C). We also manipulated the sensorimotor delay τ by adding a variable delay Δτ to the visual feedback. Again, the model quantitatively captures the decrease in frequency of the self-sustained oscillation with increasing Δτ (*SI Appendix*, section 2D). Notably, for large additional delays (∼0.5 s), the oscillations become highly regular in both amplitude and frequency, as expected for a limit cycle, suggesting that the gain μ has crossed the bifurcation threshold.

### Adaptive Responsiveness Prevents Delay-Induced Instability.

The three dynamical regimes illustrated in Fig. 4*A* highlight the challenge that the fish need to overcome: they must operate at large enough gain μ=kατ to swiftly reach the target speed, but keep clear of the bifurcation threshold beyond which oscillations become unstable. This seems challenging considering that μ is proportional to the feedback gain α, a parameter that can vary rapidly over a wide range and cannot be directly inferred from visual cues.

As its value governs the system dynamics, we sought to evaluate μ across experimental conditions, i.e., for different values of $\omega_{\text{ext}}$, α, and the additional feedback delay Δτ. Since the delay τ was found to be invariant, we estimated the responsiveness k through linear regression between $\dot{V}\left(t\right)$ and ω(t−τ).

Assuming the responsiveness k to be fixed, the dimensionless gain μ should be proportional to both α and τ. In contrast, we found that μ is quasi-independent of α (except for target speeds approaching the upper physiological limit) and increases sublinearly with both Δτ and $\omega_{\text{ext}}$ (Fig. 4*B*). These observations suggest that the responsiveness k is adaptive. Interestingly, across most of the range of parameters tested, such adaptation leads the fish to operate within the intermediate dynamic regime, which optimally balances responsiveness and stability.

### Logarithmic Transformations Balance Responsiveness and Stability.

**Fig. 5.**
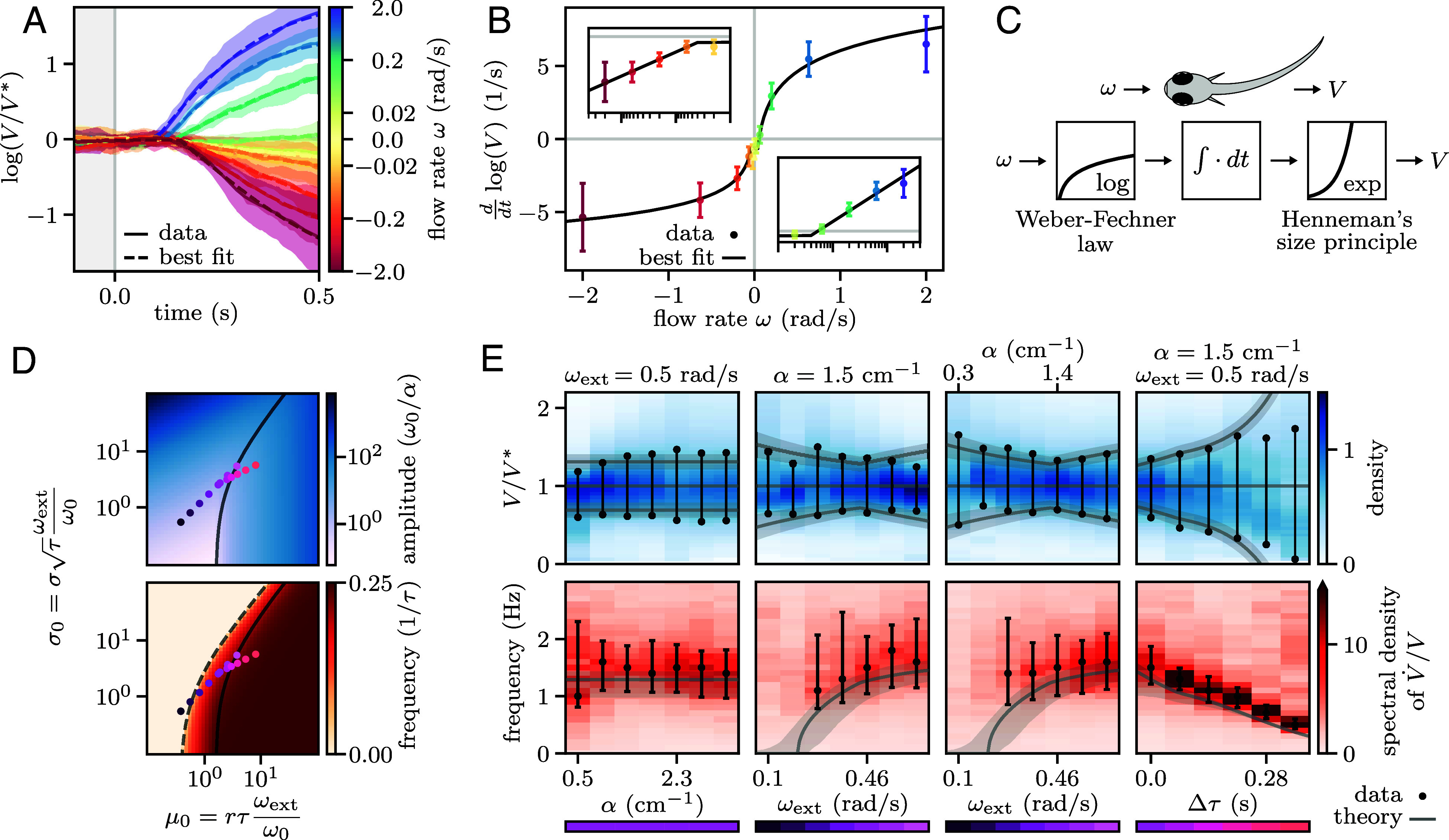
Logarithmic transformations balance responsiveness and stability. (*A*) Evolution of the swimming speed following a sudden perturbation, triggered at time t=0, of the optic flow rate ω (color-coded values). Median trajectories over trials (solid lines and shaded areas, mean and SD across N=5 fish) evolve approximately exponentially (dashed lines, best fit) after a delay τ. (*B*) Acceleration rate (extracted from *A*) as a function of ω. Beyond a threshold, the dependence is logarithmic (black line, best fit). The *Insets* show the same data on semilogarithmic scales for positive and negative ω. (*C*) Schematic showing the sequence of sensorimotor transformations. The optic flow undergoes a logarithmic compression, followed by an integration process and, finally, an exponential expansion sets the swimming speed. (*D*) Amplitude and frequency of the oscillations derived from Eq. **2** as a function of the dimensionless parameters $\mu_{0}$ and $\sigma_{0}$. There is a sharp transition to oscillatory behavior (dashed line) and a smooth transition between noise-induced and limit cycle oscillations (solid line). (*E*) *Top*: distributions of relative swimming speeds $V/V^{*}$ (blue scale). The values at half-maximum (black dots) delimit the typical range of speed fluctuations. *Bottom*: power spectra of the relative acceleration $\dot{V}/V$ (red scale) and characteristic oscillation frequency (black error bars, for peak-to-width ratios larger than 3). Data were pooled across the fish from the experiments of Fig. 4*B* (N=(33,31,28,14), from left to right). The color bars on the bottom correspond to the points in (*D*), leading to the theoretical predictions shown in gray.

To better understand the mechanism underlying the adaptive behavior, we monitored the fish transient acceleration following a sudden change in optic flow rate. After a ∼10 s period during which the fish adjusted their swimming speed to match a constant optic flow rate $\omega_{\text{ext}}$, we suddenly disabled the feedback and displayed forward or backward flows of various rates ω (Fig. 5*A*). We found that, after a delay τ, the swimming speed approximately follows an exponential evolution, with an acceleration rate $\dot{V}/V$ independent of both $\omega_{\text{ext}}$ and α (*SI Appendix*, section 2F). For $\left|\omega \right|>\omega_{\text{th}}\approx 0.03$ rad/s, the acceleration rate was found to vary logarithmically with ω for both forward and backward flow, with the fish accelerating and decelerating, respectively. For $\left|\omega \right|<\omega_{\text{th}}$ the fish shows a slight deceleration independent of ω.

These findings can be summarized as a logarithmic compression of the sensory input, followed by a temporal integration and an exponential expansion (Fig. 5*C*), and so the swimming speed can be expressed by the following stochastic nonlinear delay equation:

[2] $$\begin{array}{c} \frac{d}{\mathrm{dt}}\log\left(V\right)=r\log^{*}\left(\omega /\omega_{0}\right){|}_{t-\tau }+\sigma \xi \end{array}$$

where $\log^{*}$ denotes the symmetrized logarithm defined as $\log^{*}\left(x\right)=\text{sgn}\left(x\right)\log\left(1+|x|\right)$, a functional form that offers a reasonable approximation of the measured response function (Fig. 5*B*). The response parameters r and $\omega_{0}$ were inferred to be r=1.6±0.3 s^−1^ and $\omega_{0}=0.07\pm 0.03$ rad/s from fitting the data of Fig. 5*B*. A white noise source ξ with amplitude σ was also introduced to account for the additive noise observed in the dynamics of log(V), in agreement with the motor control literature (21). From the variability in the stabilization to constant currents, we estimated the noise amplitude at σ=0.9±0.2 s^−1/2^ (*Materials and Methods*). Importantly, the three parameters r, $\omega_{0}$ and σ were found to be independent of the experimental conditions and weakly variable across individuals.

To better interpret Eq. **2**, we can define the effective responsiveness as $k_{\text{e}}=\dot{V}{/\omega |}_{t-\tau }=r\;V{{|}_{t}\left[\log^{*}\left(\omega /\omega_{0}\right)/\omega \right]|}_{t-\tau }$. For small deviations around the target speed $V≃\omega_{\text{ext}}/\alpha$, the effective gain becomes $\mu_{\text{e}}=k_{\text{e}}\;\alpha \;\tau ≃r\;\omega_{\text{ext}}\;\tau \;\left[\log^{*}\left(\omega /\omega_{0}\right)/\omega \right]{|}_{t-\tau }$. Hence, the exponential expansion results in $\mu_{\text{e}}$ being independent of α and proportional to $\omega_{\text{ext}}$. Furthermore, because of the logarithmic compression of the optic flow rate, $k_{\text{e}}$ keeps decreasing as the swimming speed deviates from the target, preventing the oscillations from diverging.

As numerical simulations of Eq. **2** quantitatively reproduced the experimentally observed speed oscillations (*SI Appendix*, section 2I), we developed an effective linear theory to get an analytical understanding of the system’s behavior (*Materials and Methods*). In the limit of small oscillations, for which $\frac{d}{\mathrm{dt}}\log\left(V\right)≃\dot{V}/V^{*}$, we found that the dynamics are fully determined by the dimensionless gain $\mu_{0}=r\tau \omega_{\text{ext}}/\omega_{0}$ and noise amplitude $\sigma_{0}=\sigma \sqrt{\tau }\omega_{\text{ext}}/\omega_{0}$. We derived theoretical predictions for the amplitude and characteristic frequency of the fluctuations by combining the contributions of noise and nonlinearity (Fig. 5*D*). The deterministic dynamics (i.e., in the absence of noise) give rise to limit cycle oscillations for $\mu_{0}>\pi /2$, whose amplitude corresponds to an effective responsiveness $k_{\text{e}}$ close to the stability boundary $\mu_{\text{e}}=\pi /2$. The presence of noise drives the system away from the deterministic attractor, leading to larger values of |ω| and correspondingly smaller values of $k_{\text{e}}$. As in the analysis of Eq. **1**, we found a sharp transition to oscillatory dynamics for $\mu_{\text{e}}=1/e$. However, the noise tends to smooth out the bifurcation to limit cycle oscillations at $\mu_{0}=\pi /2$ (*SI Appendix*, section 2H).

While the value of α has no effect on the behavior of the solutions, increasing $\omega_{\text{ext}}$ or τ moves the system from the noise-dominated regime to the limit cycle-dominated regime with the oscillation frequency transitioning from 0 to 1/(4τ). The relative amplitude of the fluctuations depends only weakly on $\omega_{\text{ext}}$ but it is strongly modulated by τ. Plugging in our estimates of τ, σ, r, and $\omega_{0}$, we obtained theoretical predictions that quantitatively match the amplitude and frequency of the measured speed fluctuations (Fig. 5*E*). We also found that the predicted effective responsiveness matches our naive estimates through linear regression (Fig. 4*B*).

### Logarithmic Coding in the Brain.

The behavioral experiments allowed us to establish the sensorimotor operations that govern speed stabilization (Fig. 5*C*). We next asked whether these nonlinear transformations could be reflected in the neural coding of optic flow and swimming speed. To address this, we leveraged the small size and transparency of danionella’s brain to perform brain-wide functional recordings in 2-wk-old transgenic larvae expressing the GCaMP6s calcium indicators in all neurons. The reporter expression was confined to the neuronal nuclei and we used a light sheet microscope with confocal line detection in order to mitigate possible cross-talk (*Materials and Methods*) (22, 23).

**Fig. 6.**
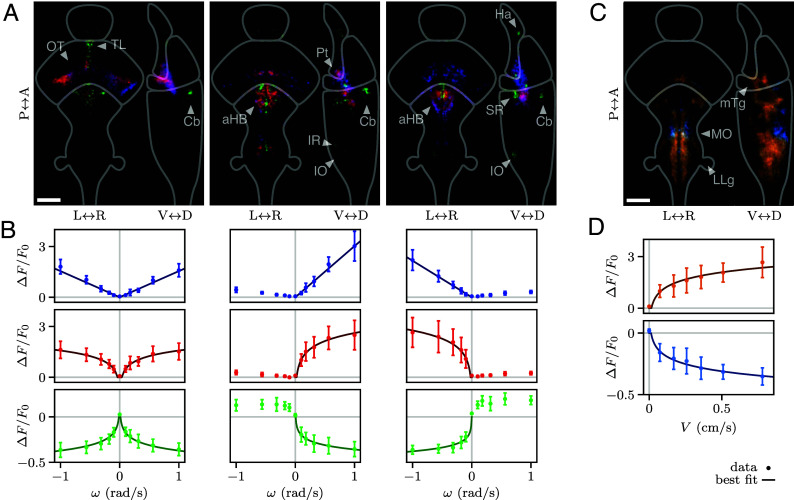
Neural activity encodes optic flow and swimming speed logarithmically. (*A*) Spatial distributions of neurons whose activity is correlated to optic flow rate ω, either to both forward and backward flow (*Left*), only forward (*Middle*), or only backward (*Right*). Blue (correlated) populations show a linear response to optic flow rate, whereas red (correlated) and green (anticorrelated) are logarithmically tuned. The density distributions are averaged over N=8 fish and integrated along different axes to obtain the projections in the horizontal (*Left*) and sagittal (*Right*) planes. They are separately normalized for each neuronal population. The outlines indicate the forebrain, midbrain, and hindbrain (from top to bottom). (OT) optic tectum; (TL) torus longitudinalis; (Cb) cerebellum; (aHB) anterior hindbrain; (Pt) pretectum; (IR) inferior raphe; (IO) inferior olive; (Ha) habenula; (SR) superior raphe. (Scale bar, 100µm.). (*B*) Tuning curves for the populations shown in (*A*), showing the average activity of each population as a function of ω (error bars, mean and SD across N=8 fish) and the best fits (solid lines). (*C*) Same as (*A*), but for neurons encoding the swimming speed V, either increasing (orange) or decreasing (light blue) logarithmically. (MO) medulla oblongata; (mTg) medial tegmentum; (LLg) lateral line ganglia. (*D*) Same as (*B*), but for the average activity of the populations shown in (*C*) as a function of V. The solid lines show the logarithmic fits.

After neuronal segmentation and signal extraction, we obtained, for each neuron, a time-trace of the relative variation in fluorescence $\Delta F/F_{0}$. Although we could monitor calcium activity in closed-loop settings (Movie S4), we mostly used open-loop experiments as they allowed us to disentangle visually driven from motor-related activity. The fish were exposed to forward and backward flows of varying rates and we simultaneously recorded their tail movements. Visually driven neurons were categorized based on their correlation (positive or negative) with forward and/or backward flow rates (Fig. 6 *A* and *B*, *Materials and Methods*). Similarly, motor-related neurons were either positively or negatively correlated with swimming speed (Fig. 6 *C* and *D*). These distinct populations displayed stereotypical spatial distributions (Fig. 6 *A* and *C*, *SI Appendix*, Fig. S12, and Movies S5 and S6).

In line with previous observations made in zebrafish (24, 25), cells positively correlated with optic flow were found in the optic tectum, pretectum, and anterior hindbrain (Fig. 6*A*). We also identified neuronal populations anticorrelated with optic flow in the habenula, torus longitudinalis, cerebellum, superior and inferior raphe, and inferior olive. We further categorized these neurons based on the shape of their tuning curves (Fig. 6*B*). We observed that, as the information driving the optomotor response propagates from the visual regions to the hindbrain, the tuning undergoes a transformation from mostly linear to logarithmic coding. We checked that these nonlinear response curves could not be accounted for by a saturation of the calcium sensor (*SI Appendix*, section 3F).

Neuronal populations encoding the swimming speed were distributed throughout the hindbrain (Fig. 6*C*), in regions consistent with known premotor areas in zebrafish (26, 27), the medulla oblongata and the medial tegmentum. We found neurons anticorrelated with swimming speed in the habenula, optic tectum, dorsal medulla, superior and inferior raphe, and lateral line ganglia. All motor-related neurons were logarithmically tuned, either positively and negatively, to the swimming speed (Fig. 6*D*).

In summary, the tuning curves of the neuronal populations engaged in visuomotor processing reveal nonlinear transformations along the visuomotor pathways that are consistent with the behavioral model.

## Discussion

We experimentally probed the sensorimotor computation at play during visually driven speed regulation in danionella, by systematically manipulating the sensory feedback. This allowed us to infer a simple mathematical model that captures the speed of the animal, including the sustained oscillations induced by the sensorimotor delay, across a wide range of experimental conditions. Our analysis reveals that the nonlinear transformations that occur at both sensory and motor ends prevent instability and enable efficient stabilization.

These sensorimotor operations, inferred from behavioral assays, were evident at the neural level in the tuning curves of visually driven and motor-associated neurons. They are also biologically grounded, reflecting two well-established principles in neuroscience (Fig. 5*C*). The sensory compression seen in our data is an instance of the Weber–Fechner law, which states that the perceived intensity of a stimulus increases logarithmically beyond a certain detection threshold (28). The expansion observed at the motor end corresponds to Henneman’s size principle (29), which states that as the motor command increases, progressively larger motor units are recruited, resulting in an exponential transformation from motor command to muscle force (30).

Our model focuses on the regulation of forward speed during continuous swimming. A natural extension would be to incorporate orientation control, which maintains the fish aligned with the external current. In addition, a more complete model should include swimming initiation, which may be triggered by the detection of optic flow (31), as well as termination, which may result from sustained swimming at either boundary of the physiological speed range.

Our results suggest that the mechanism used by the fish to stabilize their position is based on the sole perceived optic flow, and does not involve a forward internal model. Under natural conditions, fish do not only stabilize their position: internal drives and competing stimuli can override the optomotor response, allowing them to move across the environment. Our study does not rule out the possibility that internal models are at play in such general contexts, where they could prove advantageous by helping fish disentangle the consequences of their own actions from external environmental dynamics. Consistent with this idea, insects have been shown to use efference copies to modulate optic flow detection during active displacements, but not during stabilization (32–34).

The observed speed modulations are reminiscent of the self-generated oscillatory movements observed in animal locomotion, which were shown to play a role in active sensing and depth estimation (35–38). However, in our experiments, the oscillations are fully accounted for by delayed feedback control, and can be manipulated in a deterministic way through perturbations of feedback delay and optic flow rate. These observations indicate that they are a by-product of the delays in the sensorimotor loop rather than being driven by neural oscillators.

Zebrafish has been an important model system to study visuomotor control in vertebrates. Although the capacity of this animal to adapt its response to changes in the feedback gain is now well documented, the control mechanism at play, and notably the existence of an internal model, remains debated (39–42). The difficulty in reaching a definitive conclusion arises from the intermittent nature of zebrafish locomotion, which obscures the underlying sensorimotor computation. Given the close morphological and genetic proximity between danionella and zebrafish, and the existence of a comparable sensorimotor delay in both species (41), it seems likely that they share the same control mechanism. In the case of zebrafish however, the integrated optic flow rate is not directly reflected in the swimming speed, but we expect it to be encoded in the persistent activity of neuronal circuits that modulate the intensity, frequency, and duration of swimming bouts (43, 44).

Logarithmic coding has been shown to be optimal from an information-theoretical perspective as it minimizes relative error (30, 45–47), but we are not aware of any prior work addressing its role in sensorimotor control. Because it relies on fundamental neural encoding principles, we expect the control mechanism identified in danionella to be relevant across other species and sensorimotor systems in which responsiveness and stability need to be balanced. These include flight control in insects (48, 49), ocular pursuit in monkeys (50), motor control in humans (51) and position stabilization in electric fish (52), all examples in which latency-induced speed oscillations have been observed. Beyond biology, the proposed mechanism could also inspire the development of robust control strategies for robots navigating unpredictable environments (53).

## Materials and Methods

### Virtual Reality Experiments.

All experiments were performed on larvae of *D. cerebrum* of age ≈14 d post fertilization and total body length ≈4.5 mm (*SI Appendix*, section 1A). The experimental procedures were approved by the ethics committee “Le Comité d’Éthique pour l’Expérimentation Animale Charles Darwin C2EA-005.” Imaging experiments were performed using the transgenic line *Tg(elavl3:H2B-GCaMP6s)* (15), while behavioral experiments were also performed on wild type fish. The fish were immobilized in a 2% agarose gel (*SI Appendix*, section 1B) and placed in a custom-made tank (*SI Appendix*, section 1C). A Python program was written to compute the fictive velocity from the recorded tail movements and update the projected visual pattern in real-time (*SI Appendix*, section 1E). We used fluid dynamics to estimate the thrust and drag acting on the fish and computed its fictive forward speed V and angular velocity Ω (*SI Appendix*, section 1D). We calibrated these estimates using recordings of freely swimming fish. We projected a random pattern with a characteristic length scale of 5 mm on the bottom of the tank at a distance of h=5 mm from the fish. To restore visual feedback, we translated the pattern opposite to the fish heading direction with speed αVh and rotated it with angular velocity βΩ. To simulate an external current, we translated the pattern with speed $\omega_{\text{ext}}h$ along a direction that rotated together with the pattern, corresponding to a fixed direction in the virtual environment (*SI Appendix*, section 1E). To study the response to constant currents, we presented currents with different parameter values in a randomized order, with each value presented in two trials of 30 s duration.

### Impulse Response Estimation Via Reverse Correlation.

We presented fish with an external current of five minutes duration with a fluctuating flow rate of the form $\omega_{\text{ext}}\left(t\right)={\overset{¯}{\omega }}_{\text{ext}}+\sigma_{\text{ext}}\xi \left(t\right)$, where ξ is a Gaussian white noise. Then, the impulse response function $\dot{G}$ was estimated by computing the cross-correlation (20) (*SI Appendix*, section 2B):

[3] $$\begin{array}{c} \dot{G}\left(t\right)=\frac{1}{\sigma_{\text{ext}}^{2}}{\langle \left(\omega_{\text{ext}}\left(t^{\prime }-t\right)-{\overset{¯}{\omega }}_{\text{ext}}\right)\dot{V}\left(t^{\prime }\right)\rangle }_{t^{\prime }} \end{array}$$

Where we averaged over the periods of time where the fish were swimming. The impulse response could be reproduced (red line in Fig. 3*B*) by considering the first positive peak (yellow area in Fig. 3*B*, Gaussian fit) to be the response kernel K and numerically integrating the equation $\dot{V}=K*\omega$.

### Analysis of Linear Delay Equation.

We nondimensionalized Eq. **1** by considering $x\left(t^{\prime }\right)=V\left(t\right)-\omega_{\text{ext}}/\alpha$ and $t^{\prime }=t/\tau$, leading to (*SI Appendix*, section 2A):

[4] $$\begin{array}{c} \frac{\mathrm{dx}}{dt^{\prime }}{=-\mu x|}_{t^{\prime }-1}. \end{array}$$

Looking for a solution in the form of an exponential with complex growth rate λ, we obtain the following characteristic equation:

[5] $$\begin{array}{c} \lambda =-\mu e^{-\lambda }, \end{array}$$

whose solutions are given by the Lambert W function as $\lambda_{l}=W_{l}\left(-\mu \right)$. The behavior is dominated by the largest growth rate $\text{Re}(W_{0}\left(-\mu \right))$, with the corresponding oscillation frequency $\text{Im}(W_{0}\left(-\mu \right))/\left(2\pi \right)$.

### Responsiveness Estimation Via Linear Regression.

We extracted the slope of the relationship between ω(t−τ) and $\dot{V}$ using the Theil-Sen estimator to reduce the effect of outliers (54) (*SI Appendix*, section 2E). We used the estimate of τ obtained from the impulse response and considered the data from the experiments where the fish were swimming against constant currents.

### Open-Loop Perturbations.

We presented fish with constant currents in closed-loop and then suddenly presented a certain optic flow rate ω in open-loop for a duration of 0.5 s. The different values of ω were presented in a randomized order with each value presented at least 10 times for each one of several baseline closed-loop conditions $\left(\omega_{\text{ext}},\alpha \right)$ (*SI Appendix*, section 2F). The evolution of the speed following the perturbation was fit with a delayed exponential evolution:

[6] $$\begin{array}{c} V\left(t\right)=V_{0}\exp\left[H\left(t-t_{0}\right)A\tanh\left(\frac{\lambda }{A}\left(t-t_{0}\right)\right)\right], \end{array}$$

where H is the Heaviside step function and we included a saturation in the form of a hyperbolic tangent. The dependence of the acceleration rate λ on the flow rate was fit with a logarithm:

[7] $$\begin{array}{c} \lambda \left(\omega \right)=\lambda_{0}\pm \lambda_{\pm }\log\left(\pm \omega /\omega_{\text{th}}\right), \end{array}$$

respectively, for positive and negative values of ω.

### Noise Amplitude Estimation.

We estimated the noise amplitude by computing the SD of the increments of the speed on a logarithmic scale (*SI Appendix*, section 2G):

[8] $$\begin{array}{c} \sigma =\sqrt{\langle \frac{{\left(\log\left(V_{t+\Delta t}\right)-\log\left(V_{t}\right)\right)}^{2}}{\Delta t}\rangle }. \end{array}$$

For the estimate we considered the data from the experiments where the fish were swimming against constant currents.

### Analysis of Stochastic Nonlinear Delay Equation.

We nondimensionalized Eq. **2** in the limit of small oscillations by considering $x\left(t^{\prime }\right)=\left(\omega_{\text{ext}}-\alpha V\left(t\right)\right)/\omega_{0}$ and $t^{\prime }=t/\tau$, leading to (*SI Appendix*, section 2H):

[9] $$\begin{array}{c} \dot{x}=-\mu_{0}\log^{*}\left(x{|}_{t^{\prime }-1}\right)+\sigma_{0}\xi^{\prime }{|}_{t^{\prime }}, \end{array}$$

where $\xi^{\prime }$ denotes delta-correlated noise in the dimensionless time $t^{\prime }$. For a certain value of ${x|}_{t^{\prime }-1}=x_{0}$ the system evolves locally with a rate of change $-\mu_{0}\log^{*}\left(x_{0}\right)$ which would equivalently arise from a linear system with a gain $\mu_{\text{e}}=\mu_{0}\log^{*}\left(x_{0}\right)/x_{0}$. We obtained an estimate of the amplitude of limit cycle oscillations $A_{\text{lc}}$ by considering the value of $x_{0}$ for which the effective gain becomes π/2:

[10] $$\begin{array}{c} A_{\text{lc}}=-\frac{2\mu_{0}}{\pi }W_{-1}\left(-\frac{\pi }{2\mu_{0}}\exp\left(-\frac{\pi }{2\mu_{0}}\right)\right)-1. \end{array}$$

The noise term induces fluctuations with an amplitude $A_{\text{n}}$ that can be derived from the following implicit relation:

[11] $$\begin{array}{c} A_{\text{n}}\log\left(1+A_{\text{n}}\right)=\frac{\sigma_{0}^{2}}{2\mu_{0}c_{\text{n}}^{2}}, \end{array}$$

which we obtained using the fact that a Ornstein–Uhlenbeck process with gain $\mu_{\text{e}}$ and noise amplitude $\sigma_{0}$ leads to a fluctuations with SD $c_{\text{n}}A_{\text{n}}=\sigma_{0}/\sqrt{2\mu_{\text{e}}}$ (55). By combining the limit cycle and the noise-induced amplitudes we obtained an estimate of the total amplitude of the fluctuations and thus of the effective gain $\mu_{\text{e}}\left(\mu_{0},\sigma_{0}\right)$. The predicted oscillation frequency is then given by $\text{Im}\left(W_{0}\left(-\mu_{\text{e}}\right)\right)/\left(2\pi \right)$.

### Brain Imaging Experiments.

We used a custom-made light sheet microscope with confocal slit detection to excite and record the fluorescence across the brain of the fish (*SI Appendix*, section 3A). We recorded stacks of 25 different layers at a rate of ≈2.3 brain volumes per second for a laser power of ≈150 µW. For the open-loop experiments we presented constant currents with different values of flow rate ω in a randomized order, with each value presented in 3 trials of 20 s duration (*SI Appendix*, section 3B).

### Processing of Brain Images.

We corrected an eventual horizontal shift in the images by computing the cross-correlation between different images (*SI Appendix*, section 3C). Then, we computed the average covariance between the intensity of neighboring pixels over the recording. Applying a blob detection algorithm to the resulting images gave the positions of all the neurons showing a modulation in activity during the recording. Then, we computed the relative fluorescence change $\Delta F/F_{0}$ for each neuron taking as baseline $F_{0}$ the median fluorescence in the absence of optic flow and tail movements.

### Registration to a Reference Brain.

To compare the results for different fish, we acquired a stack of brain images with high vertical resolution and long exposure for each fish. Then, we numerically estimated the optimal affine transform between different stacks by maximizing their cross-correlation through gradient descent (*SI Appendix*, section 3D). The reference stack was obtained by averaging the aligned stacks for all fish and then mapping the left–right symmetry plane to the middle plane of the stack.

### Correlation Analysis.

We identified the neurons whose activity is significantly correlated or anticorrelated with four different signals (*SI Appendix*, section 3E): flow rate |ω|, forward flow rate ωH(ω), backward flow rate −ωH(−ω), and swimming speed V. We computed the Pearson correlation between each of these signals and the relative fluorescence change $\Delta F/F_{0}$ for every neuron. To determine significantly high correlation values, we built null distributions by computing the correlation with time-shifted versions of the signals with an autocorrelation smaller than 0.25 in magnitude. To account for the problem of multiple comparison for hypothesis testing, we used the Benjamini–Hochberg procedure (56), controlling the false discovery rate with a significance threshold of $10^{-5}$. Neurons present in more than one out of the 8 identified populations were assigned to the population for which the correlation was largest in magnitude. To separate the neurons correlated to optic flow into subpopulations showing linear or logarithmic coding, we computed the correlation values with the logarithm of the corresponding signal and compared them with the previously computed values. We fitted the tuning curves either with a linear response Ax or a logarithmic one $A\log\left(x/x_{0}\right)H\left(x-x_{0}\right)$ as a function of the corresponding signal x. To visualize the spatial distributions of the neuronal populations, we used kernel density estimation with a uniform spherical kernel of radius 5 µm. We assigned a color to each pixel by computing the weighted sum of the hues corresponding to each population with the respective value of the density. We separately normalized each density to result in a maximum pixel intensity of 1, and the weighted sum was renormalized when any color channel exceeded 1. We used the Max Planck Zebrafish Brain (mapzebrain) atlas (57) to identify the brain regions associated with the neuronal populations.

## Supplementary Material

Appendix 01 (PDF)

Movie S1. Example of a fish actively turning in the virtual reality to align to external currents oriented in different directions. The movie shows the visual stimulus presented to the fish (top, left), the video recording of the tail with overlaid segmentation (top, middle), a schematic of the fish orientation in the virtual environment, with the fish velocity vector in black and the external current orientation in gray (top, right), and the traces of flow rates and orientations relative to fish and current (bottom). *ω*_ext_ = 1 rad/s, *α* = 6 cm^−1^, *β* = 1. $\theta_{\text{ext}}=\theta_{\text{fish}}^{\text{start}}+90^{\circ }$ for the first trial and $\theta_{\text{ext}}=\theta_{\text{fish}}^{\text{start}}-90^{\circ }$ for the second.

Movie S2. Example of a fish swimming in the virtual reality and stabilizing its position against forward-directed currents with different flow rates. The movie shows the visual stimulus presented to the fish (top, left), the video-recording of the tail with overlaid segmentation (top, right), and the traces of flow rates relative to fish and current (bottom). *α* = 6 cm^−1^, *β* = 1. *ω*_ext_ = 1.5 rad/s for the first trial and *ω*_ext_ = 0.67 rad/s for the second.

Movie S3. Example of a fish swimming in the virtual reality to stabilize external currents for different values of the feedback gain. The movie shows the visual stimulus presented to the fish (top, left), the video-recording of the tail with overlaid segmentation (top, right), and the traces of flow rates and speeds relative to fish and current (bottom). *ω*_ext_ = 1 rad/s, *β* = 1. *α* = 9 cm^−1^ for the first trial and *α* = 4 cm^−1^ for the second.

Movie S4. Example of the brain activity of a fish while it stabilizes against external currents in the virtual reality. The movie shows the recorded sequences of 4 different sections of the brain (top) and the traces of flow rates relative to fish and current (bottom). Time is sped up by a factor of 10. *α* = 1.5 cm^−1^, *β* = 0.25, *ω*_ext_ = (0.22, 0.33, 0.5) rad/s for the different trials.

Movie S5. Visualization of the neuronal populations identified through correlation analysis. The movie shows the three-dimensional spatial distribution of the identified neuronal populations (average densities corresponding to the projections shown in the main text) mapped onto the same reference stack. The densities are normalized so that a single neurons contributes a fraction of 0.1 of the maximum hue intensity. The densities are overlaid on top of the reference stack, shown in grayscale. The movie shows the densities layer by layer moving sequentially from the dorsal side to the ventral side.

Movie S6. Visualization comparing the neuronal populations correlated with swimming speed and optic flow in different directions. Analogous to movie S5, but the populations are shown together (independently of positive or negative and linear or logarithmic tuning) to compare the spatial distribution of populations correlated with different signals.

## Data Availability

Behavioral and neural data and code for experiments and analysis have been deposited in Zenodo (https://doi.org/10.5281/zenodo.15187167) (58).
